# Free Energy, Enthalpy and Entropy from Implicit Solvent End-Point Simulations

**DOI:** 10.3389/fmolb.2018.00011

**Published:** 2018-02-08

**Authors:** Federico Fogolari, Alessandra Corazza, Gennaro Esposito

**Affiliations:** ^1^Dipartimento di Scienze Matematiche, Informatiche e Fisiche, Universita' di Udine, Udine, Italy; ^2^Istituto Nazionale Biostrutture e Biosistemi, Rome, Italy; ^3^Dipartimento di Area Medica, Universita' di Udine, Udine, Italy; ^4^Science and Math Division, New York University Abu Dhabi, Abu Dhabi, United Arab Emirates

**Keywords:** free energy, enthalpy, entropy, molecular dynamics simulations, implicit solvent, continuum solvent, MM/GBSA

## Abstract

Free energy is the key quantity to describe the thermodynamics of biological systems. In this perspective we consider the calculation of free energy, enthalpy and entropy from end-point molecular dynamics simulations. Since the enthalpy may be calculated as the ensemble average over equilibrated simulation snapshots the difficulties related to free energy calculation are ultimately related to the calculation of the entropy of the system and in particular of the solvent entropy. In the last two decades implicit solvent models have been used to circumvent the problem and to take into account solvent entropy implicitly in the solvation terms. More recently outstanding advancement in both implicit solvent models and in entropy calculations are making the goal of free energy estimation from end-point simulations more feasible than ever before. We review briefly the basic theory and discuss the advancements in light of practical applications.

## 1. Introduction

Free energy is the key quantity in the description of the thermodynamics of biological systems, and therefore an important objective of biomolecular simulations. In spite of its relevance, free energy calculations are *not* performed for every simulation performed although most often concepts like (local) stability are used to describe the results of the simulation suggesting a thermodynamic description.

The difficulties arise obviously from the calculation of the entropy of the system which cannot be immediately computed as an ensemble average over simulation snapshots, contrary to the enthalpy. The free energy *G* can be formally related to an ensemble average:

(1) $$\begin{array}{r} G=RT\log\langle \exp\left(\beta U\right)\rangle \end{array}$$

where *R* is the gas constant, *T* the temperature, β is equal to $\frac{1}{RT}$, *U* is the potential energy and ⟨⟩ indicates thermodynamic ensemble average. However, as pointed out many years ago (Beveridge and DiCapua, 1989), this formula is of no practical use unless all configurational space enters (implicitly or explicitly) the average, because lowest probability configurations display the highest value contribution to the average and there cannot be convergence for upper unbound potentials. In molecular simulations typically only near-equilibrium configurations are sampled.

For this reason free energy differences calculation is performed using methods, like umbrella sampling (Torrie and Valleau, 1977), thermodynamic integration (Straatsma and McCammon, 1991) or metadynamics (Laio and Parrinello, 2002), that compute by various methods the free energy along a pathway connecting end-point states, even if it is often just the difference in free energy between the latter points that is actually needed.

If the two end-points are close enough a simulation of one or both end-points may be performed and free energy differences obtained by free energy perturbation (Zwanzig, 1954), but this is not possible in general.

The problem with the computation of the entropy from a single MD simulation is that only part of the configurational space is accessed by simulation.

In this short perspective we remark that the long sought calculation of free energies from end point simulations may be afforded with reasonable accuracy from implicit solvent end-point simulations. In particular, some recent developments in the way entropy is calculated in fact allow to compute free energy from a single simulation, so it is worth to review briefly the theory here and to provide simple formulae to compute free energy, entropy and enthalpy from implicit solvent simulations.

## 2. Theory

We consider here simulations sampling different states of a system *A* in solution. This can be done by different simulations if the states are kinetically well separated, or by post-processing a single simulation to divide microstates belonging to different states.

If the simulation is extensive enough most probable microstates are sampled and representative thermodynamic ensembles are generated for the different states which include samples of all microstates whose probability density is higher than a threshold.

Enthalpy can be computed as the energy ensemble average, whereas entropy is problematic, in particular for what concerns solvent degrees of freedom. Although solvent entropy has been taken into account in some studies (e.g., De Simone et al., 2005) the extensive correlation among solvent molecules makes solvent entropy estimation a difficult task. In order to get rid of this problem we treat the system using an implicit solvent model.

### 2.1. Implicit solvent models

Following the excellent reviews available (Gilson et al., 1997; Roux and Simonson, 1999; Wereszczynski and McCammon, 2012) we write the standard chemical potential, or molar free energy, in the following form:

(2) $$\begin{array}{r} G_{A}^{0}=-RT\log\left(\frac{\int \exp\left(-\beta U\left({\vec{r}}_{A},{\vec{r}}_{S}\right)\right)d{\vec{r}}_{A}d{\vec{r}}_{S}}{\int \exp\left(-\beta U\left({\vec{r}}_{S}\right)\right)d{\vec{r}}_{S}}\right)+C \end{array}$$

where ${\vec{r}}_{A}$ and ${\vec{r}}_{S}$ are the solute and solvent coordinates. *C* includes the integrals over the reference state (1M, random orientation) and the momentum integrals (independent of conformation) that cancel when comparing different states of the same system and the term $P^{0}{\bar{V}}_{A}$, with *P*^0^ the standard pressure and ${\bar{V}}_{A}$ the partial volume of the solute, whose dependence on conformation provides negligible effects at standard pressure. For a derivation see Gilson et al. (1997).

For this reason in the following *C* will not be considered further.

Due to the difficulties in estimating the entropy of highly correlated solvent molecules the solvation potential of mean force ($\Delta W\left({\vec{r}}_{A},T\right)$) is defined by integrating out the solvent degrees of freedom.

(3) $$\begin{array}{r} \exp\left(-\beta \Delta W\left({\vec{r}}_{A},T\right)\right)=\frac{\int \exp\left(-\beta \left(U_{AS}\left({\vec{r}}_{A},{\vec{r}}_{S}\right)+U_{S}\left({\vec{r}}_{S}\right)\right)\right)d{\vec{r}}_{S}}{\int \exp\left(-\beta U_{S}\left({\vec{r}}_{S}\right)\right)d{\vec{r}}_{S}} \end{array}$$

where *U*_*AS*_ denotes the energy terms that couple the solute and the solvent and *U*_*S*_ is the solvent energy.

With this definition and the assumptions made above we have:

(4) $$\begin{array}{r} G_{A}^{0}=-RT\log\left(\int \exp\left(-\beta \left(U\left({\vec{r}}_{A}\right)+\Delta W\left({\vec{r}}_{A},T\right)\right)\right)d{\vec{r}}_{A}\right) \end{array}$$

where the dependence on the temperature of the solvation energy Δ*W* has been made explicit.

Implicit solvent models provide a functional form and parameters for $\Delta W\left({\vec{r}}_{A},T\right)$.

Although the implicit solvent model based on the Poisson-Boltzmann equation (Fogolari et al., 2002) and the solvent accessible surface area (PBSA) could be used in molecular dynamics simulations (Fogolari et al., 2003), the method based on the Generalized Born model and solvent accessible surface area (GBSA) is the method of choice for its computational efficiency (Still et al., 1990; Onufriev et al., 2004). Limitations of both methods include treatment of small crevices and cavities, where water could not display bulk solvation properties, and neglection of curvature dependence of surface tension coefficient (Nicholls et al., 1991). For GBSA an additional limitation could be due to the dependence of empirical parameters on molecular shape (see e.g., Fogolari et al., 2015a). Compared to approaches simulating the solutes in explicit solvent and post-processing the trajectory in implicit solvent (Kollman et al., 2000), methods that generate the configurational ensemble using the same implicit solvent used for energy calculations are more consistent because they don't suffer from possible mismatches between the implicit and explicit solvent models.

In recent years some of the available implicit solvent models (including forcefield and set of parameters used (Swanson et al., 2005, 2007)) have been shown to be extremely accurate in the treatment of protein thermodynamics, as demonstrated by the study by Simmerling and coworkers, where 16 out of 17 proteins could be correctly folded using the GB-Neck2 implicit solvent model and for 14 of them the native fold was preferred over the misfolded one (Mongan et al., 2007; Nguyen et al., 2014).

### 2.2. Enthalpy in implicit solvent models

The molar enthalpy is obtained as:

(5) $$\begin{array}{r} H_{A}^{0}=G_{A}^{0}+TS_{A}^{0}=G_{A}^{0}-T\frac{\partial G_{A}^{0}}{\partial T} \end{array}$$

When the derivation is performed, taking into account that also $\Delta W\left({\vec{r}}_{A},T\right)$ depends on the temperature, we obtain:

(6) $$\begin{array}{r} H_{A}^{0}=<U+\Delta W>-T<\frac{\partial \Delta W}{\partial T}> \end{array}$$

where the symbol <> indicates the ensemble average of the quantity within brackets, i.e., the average over the simulation of the same quantity.

The above equation shows that the enthalpy, which is obtained as the energy ensemble average in MD simulations, is directly related to the ensemble average of the implicit solvent model potential energy. The difference is expressed by the $-T<\frac{\partial \Delta W}{\partial T}>$ term which may however be obtained by explicit derivation with respect to the temperature of the solvation energy terms in the implicit solvation model.

In the GBSA model the solvation energy is the sum of an electrostatic (Δ*W*^*el*^) and a surface tension (Δ*W*^*SA*^) term:

(7) $$\begin{array}{r} \Delta W=\Delta W^{el}+\Delta W^{SA}=\frac{1}{2}\left(\frac{1}{\epsilon \left(T\right)}-\frac{1}{\epsilon_{in}}\right)\underset{i,j}{\sum }q_{i}q_{j}f_{ij}+\gamma \left(T\right)A \end{array}$$

where ϵ(*T*) and ϵ_*in*_ are the solvent and molecular (typically 1) dielectric constants, respectively, *q*_*i*_ is the charge of the *i*^*th*^ atom, *f*_*ij*_ a pairwise function depending on all atoms' coordinates, γ(*T*) is a surface tension and *A* is the solvent-accessible surface area. The dependence on temperature of the solvent dielectric constant and of the surface tension has been made explicit in the above equation. The contributions to the derivative of Δ*W* with respect to temperature are easily obtained from the electrostatic and surface tension solvation energy terms, when the temperature dependence of the parameters of the implicit solvent model is known:

(8) $$\begin{array}{r} \frac{\partial \Delta W}{\partial T}=\frac{\partial \Delta W^{el}}{\partial T}+\frac{\partial \Delta W^{SA}}{\partial T}\\ =\Delta W^{el}\left(\frac{\epsilon_{in}}{\epsilon \left(\epsilon -\epsilon_{in}\right)}\right)\frac{\partial \epsilon }{\partial T}+\Delta W^{SA}\frac{1}{\gamma }\frac{\partial \gamma }{\partial T} \end{array}$$

### 2.3. Entropy in implicit solvent models

The entropy is linked to the configurational solute probability distribution.

First consider that the probability density distribution in the implicit solvent model is:

(9) $$\begin{array}{r} \rho \left({\vec{r}}_{A},T\right)=\frac{\exp\left(-\beta \left(U\left({\vec{r}}_{A}\right)+\Delta W\left({\vec{r}}_{A},T\right)\right)\right)}{\left(\int \exp\left(-\beta \left(U\left({\vec{r}}_{A}\right)+\Delta W\left({\vec{r}}_{A},T\right)\right)\right)d{\vec{r}}_{A}\right)} \end{array}$$

When the derivation of the free energy is performed the entropy can be written as:

(10) $$\begin{array}{r} S_{A}^{0}=-\frac{\partial G_{A}^{0}}{\partial T} \end{array}$$

(11) $$\begin{array}{rc} = & -R\int \rho \left({\vec{r}}_{A},T\right)\log\left(\rho \left({\vec{r}}_{A},T\right)\right)d{\vec{r}}_{A}-<\frac{\partial \Delta W}{\partial T}> \end{array}$$

(12) $$\begin{array}{r} s=-R<\log\left(\rho \left({\vec{r}}_{A},T\right)\right)>-<\frac{\partial \Delta W}{\partial T}> \end{array}$$

Rewriting the entropy in terms of probability density allows to rewrite the free energy in turn as the sum of an ensemble average and a configurational entropy term, which is itself an ensemble average:

(13) $$\begin{array}{r} G_{A}^{0}=<U+\Delta W>+RT<\log\left(\rho \left({\vec{r}}_{A},T\right)\right)> \end{array}$$

As said above the difficulty in estimating free energies resides in the entropy estimation which requires consideration of both sampled and non-sampled configurational space. Similarly, to estimate $\rho \left({\vec{r}}_{A},T\right)$, consideration of non-sampled configurational space is needed. Implicit solvent models circumvent the problem treating solvent entropy implicitly, through the parameters and their temperature dependence.

In the above equation the configurational entropy is written formally as an ensemble average of the probability density. In this respect a convenient description of systems containing proteins is the bond, angle, torsion (BAT) representation (Go and Scheraga, 1976), because bonds, and to some extent also angles, contribute very little to changes in entropy for different states of proteins (e.g., notably, bound and non-bound) (Karplus et al., 1987). For this reason entropy is estimated often considering to a first approximation only torsional degrees of freedom and possibly external rotation and translation degrees of freedom.

Other descriptions, e.g., in cartesian coordinates, have been used and the entropy has been calculated assuming the system is moving harmonically or anharmonically about the energy minimum (see recent reviews Polyansky et al., 2012; Wereszczynski and McCammon, 2012). This approximation may however be poor for loops or other unrestrained parts of the molecules.

**Figure 1:**
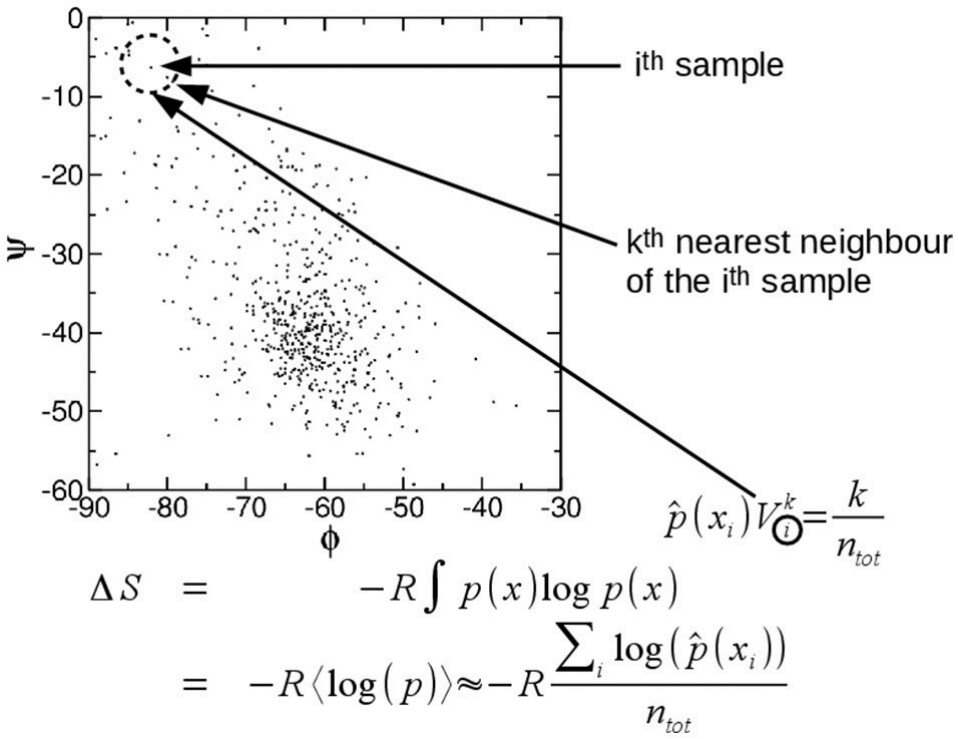
Pictorial illustration of the k-th nearest neighbor estimation of entropy. See text.

A particularly attractive method to compute entropy is the approach proposed by Singh et al. (2003) which was further developed by the same authors and others (Hnizdo et al., 2003, 2007, 2008; Darian et al., 2005; Numata et al., 2007; Wang et al., 2009; Mukherjee, 2011; Fenley et al., 2014; Huggins, 2014; Fogolari et al., 2015b, 2016). In practice the probability density ρ(*x*_1_, *x*_2_, …, *x*_*s*_) may be estimated considering a ball of radius *r*_*i*_ around each configurational sample ${\vec{x}}_{i}$ up to the k-th nearest neighbor (Figure 1), then the local probability density ($\hat{\rho }\left({\vec{x}}_{i}\right)$) is obtained by dividing the number *k* of the *n* samples which are found inside the ball over the volume of the ball *V*_*i*_ and *n*, i.e.,:

(14) $$\begin{array}{r} \hat{\rho }\left({\vec{x}}_{i}\right)=\frac{1}{V_{i}}\frac{k}{n} \end{array}$$

The idea is very simple and can be made rigorous (Singh et al., 2003). When equation 14, or its exact form, is substituted in equation 13 the free energy may be estimated easily from configurational samples.

The discrepancy between naive and exact treatment is limited. Note that the volume around each configurational sample is tuned to match the local density and there is no need consider regions where no configurational sample is found. Obviously the dimensionality of a protein system is large and it is not possible in practice to consider a probability density without actually assuming that most degrees of freedom are decoupled from each other.

The groups of Gilson (Killian et al., 2007) and Tidor (King and Tidor, 2009; King et al., 2012) have proposed treatments of mutual information that provide a practical estimate of global entropy (actually, an upper bound) considering only single degrees of freedom and pairwise mutual information. Depending on the number of samples the approach can be easily extended to higher orders of correlation.

## 3. Conclusion

We have recapitulated above the fundamentals of implicit solvent free energy calculations from end-point simulations, recalling the relationship between explicit and implicit solvent models, and showing how entropy and enthalpy can be obtained from implicit solvent simulations.

From a practical point of view we can estimate free energy from implicit solvent simulations using equation 13:

$$\begin{array}{l} G_{A}^{0}=<U+\Delta W>+RT<\log\left(\rho \left({\vec{r}}_{A},T\right)\right)> \end{array}$$

The ensemble average of *U* + Δ*W* is provided by the potential energy in the implicit solvent model used, whereas the entropy $-R<\log\left(\rho \left({\vec{r}}_{A},T\right)\right)>$ must be computed from the conformational ensemble.

The available implicit solvent models and parameters, together with forcefield parameters, have been shown to be accurate enough to reproduce complex phenomena like protein folding giving confidence in the accuracy of the implicit solvent potential of mean force. The average of the potential energy over an implicit solvent molecular dynamics trajectory thus provides the first term in the above equation.

The entropic term may be estimated using the nearest neighbor method which is emerging as an accurate entropy estimator, with many advantages over more traditional methods, including lack of hypothesis on non-sampled conformational space, no need to consider explicitly non-sampled conformational space and sound theoretical basis.

To make the application of the method straightforward we have implemented the nearest-neighbor method in two programs, PDB2ENTROPY and PDB2TRENT, available through the git-hub repository (URL: https://github.com/federico-fogolari) which allow to compute conformational and rotational-translational entropy directly from the conformational ensemble in PDB format.

In summary, the recent advancements in solvation models and entropy calculation, based on the nearest-neighbor method, are making computation of free-energy from end-point simulations significantly more accurate than before, with many possible applications in the next future.

## Author contributions

All authors listed, have made substantial, direct and intellectual contribution to the work, and approved it for publication.

### Conflict of interest statement

The authors declare that the research was conducted in the absence of any commercial or financial relationships that could be construed as a potential conflict of interest.
